# ADVANCING HEALTH EQUITY AMONG OLDER ADULTS THROUGH COMMUNITY ENGAGED RESEARCH

**DOI:** 10.1093/geroni/igad104.0750

**Published:** 2023-12-21

**Authors:** Rachel O’Conor, Marquita Lewis-Thames

## Abstract

Health equity, the absence of unfair and avoidable differences in health among social groups, has garnered mainstream attention due to recent civil unrest in communities, yet remains an elusive goal for aging health researchers. Community-engaged research is one approach to improve older adult health equity. Community-engaged research involves partnerships between researchers and community organizations, community health centers, and community members to pursue a common goal. To shift the traditional paradigm of investigator-led and -implemented research, community-engaged principles emphasize the co-production and ownership of knowledge. While community-engaged research has a successful track record in facilitating health disparities research, far fewer partnerships have been formed to 1) elevate the voices of older adults and 2) investigate and act on root causes of health disparities in older adults. To advance the practice of community-engaged research focused on older adult health, this symposium presents five community-academic partnerships elevating older adult voices from diverse groups and health conditions while simultaneously describing engagement strategies across the research process. First, Grant outlines processes for leveraging community representatives as partners in developing multilevel interventions for older cancer survivors. Second, Filec describes a multi-year community-engaged research journey that culminated with a study investigating the health impacts of a home repair program. Third, Singh details a user-centered design approach through a community advisory board to develop a telehealth navigation tool. Fourth, Lewis-Thames describes a community-based qualitative assessment capturing telehealth utilization among rural older cancer survivors. Lastly, Oladejo presents the community-driven development of a culturally-tailored dementia caregiving program for Black caregivers. This is a Community-Engaged Research Interest Group Sponsored Symposium.

